# Supplementary material for: MetaRibo-Seq measures translation in microbiomes
Source: Nat Commun. 2020 Jun 29;11:3268. doi: 10.1038/s41467-020-17081-z (PMC7324362; doi:10.1038/s41467-020-17081-z)
Supplement: Supplementary file 10 — Supplementary Data 7 [file 41467_2020_17081_MOESM10_ESM.zip › File2/Confidence_High_Taxonomy/271044_out.krona.html]

Javascript must be enabled to view this page.

members
magnitude
magnitudeUnassigned
count
unassigned
taxon
rank

271044\_out

22

superkingdom
22
2

22
976
phylum

class
200643
22

order
171549
22

815
22
family

genus
816
22

1739298
3

SRS143991\_contig\_number\_11834SRS147271\_contig\_number\_31739SRS893341\_contig\_number\_5596
species

1
477666

SRS014923\_contig\_number\_41454
species


SRS018623\_contig\_number\_3618SRS143070\_contig\_number\_12728SRS144362\_contig\_number\_14350SRS146764\_contig\_number\_92
674529
4
species

species
11
329854

SRS018623\_contig\_number\_14SRS019286\_contig\_number\_330SRS049995\_contig\_number\_675SRS049995\_contig\_number\_24757SRS056259\_contig\_number\_416SRS1041137\_contig\_number\_30347SRS142599\_contig\_number\_243SRS144362\_contig\_number\_10300SRS144362\_contig\_number\_13714SRS144362\_contig\_number\_22589SRS893369\_contig\_number\_8642

species
246787
1

SRS062427\_contig\_number\_2231


SRS147557\_contig\_number\_11918
46506
1
species


SRS013521\_contig\_number\_12554
28116
1
species
